# Preferences for active and aggressive intervention among patients with advanced cancer

**DOI:** 10.1186/1471-2407-10-592

**Published:** 2010-10-28

**Authors:** Vincent Maida, Jonathan Peck, Marguerite Ennis, Navjot Brar, Alexandria R Maida

**Affiliations:** 1 Division of Palliative Medicine, William Osler Health System, Toronto, Ontario, Canada; 2 Division of Palliative Care, University of Toronto, Toronto, Ontario, Canada; 3 Division of Palliative Care, McMaster University, Hamilton, Ontario, Canada; 4 Faculty of Science, University of Western Ontario, London, Ontario, Canada; 5 Applied Statistician, Markham, Ontario, Canada; 6 Department of Biology, University of Toronto, Mississauga, Ontario, Canada; 7 Faculty of Arts & Science, University of Toronto, Toronto, Ontario, Canada

## Abstract

**Background:**

Intrinsic to "Patient-Centered Care" is being respectful and responsive to individual patient preferences, expressed needs, and personal values. Establishing a patient's preferences for active and aggressive intervention is imperative and foundational to the development of advance care planning. With the increasing awareness and acceptance of palliative philosophies of care, patients with advanced cancer are increasingly transitioning from active and aggressive medical management (AAMM) to conservative palliative management (CPM).

**Methods:**

A cross-sectional study based on a prospective and sequential case series of patients referred to a regional palliative medicine consultative program was assembled between May 1, 2005 and June 30, 2006. Patients and/or their substitute decision makers (SDM) completed a questionnaire, at baseline, that assessed their preferences for AAMM en route to their eventual deaths. Seven common interventions constituting AAMM were surveyed: cardiopulmonary resuscitation (CPR) & mechanical ventilation (MV), chemotherapy, antibiotics, anticoagulants, blood transfusions, feeding tubes, and artificial hydration. Multivariable analyses were conducted on the seven interventions individually as well as on the composite score that summed preferences for the seven interventions.

**Results:**

380 patients with advanced cancer agreed to participate in the study. A trend to desire a mostly conservative palliative approach was noted as 42% of patients desired one or fewer interventions. At baseline, most patients and their SDM's were relatively secure about decisions pertaining to the seven interventions as the rates of being "undecided" ranged from a high of 23.4% for chemotherapy to a low of 3.9% for feeding tubes. Multivariable modeling showed that more AAMM was preferred by younger patients (P < 0.0001), non-Caucasians (P = 0.042), patients with higher baseline Palliative Performance Scale scores (P = 0.0002) and where a SDM was involved in the decision process (p = 0.027). Non-statistically significant trends to prefer more AAMM was observed with male gender (p = 0.077) and higher levels of the Charlson Comorbidity index (p = 0.059). There was no association between treatment preferences and cancer class.

**Conclusions:**

Although the majority of patients with advanced cancer in this study expressed preferences for CPM, younger age, higher baseline PPSv2, and involvement of SDMs in the decision process were significantly associated with preferences for AAMM.

## Background

The publication of the landmark SUPPORT (Study to Understand Prognoses and Preferences for Outcomes and Risks of Treatments) controlled trial in 1995 served to highlight major problems with end-of-life decision making related to weaknesses in advance care planning and less than optimal communication between physicians and their patients and families [1]. Among many findings, decreased adherence with patient preferences was demonstrated [1]. SUPPORT demonstrated greater emphasis on the provision of burdensome and ultimately ineffective/futile interventions to patients with advanced illness near the end-of-life [1]. Consequently, patients experienced poor pain and symptom management with resultant decreased quality of life [1]. SUPPORT has subsequently served as a major catalyst to improve end-of-life communication as well as promoting the development of Palliative Care as a specialty. The shortfalls identified in SUPPORT also led Steinhauser in 2000 to report on the factors considered most important by patients and their families at end-of-life: pain and symptom management, good physician-patient communication, being prepared for what to expect, achieving a sense of completion in life, clear decision-making, and being treated as a "whole person" [2]. All of these are now regarded among the core values and tenets of Palliative Care.

Patients with advanced cancer may be regarded as being in transition from active and aggressive medical management (AAMM) to conservative palliative management (CPM). Prior to the advent of palliative care, advanced cancer patients would generally continue with AAMM until their death. "AAMM" may be described and defined as consisting of all active medical treatments or active interventions that deal with physiologic derangements or medical complications experienced by the patient. The prime goal and intent of AAMM is to potentially sustain or prolong life. Hence, many authors have termed them "Life-sustaining" or "Life-prolonging" treatments or interventions. It may be argued that when successful and appropriately applied, such treatments may also improve quality of life. However, the later in the disease trajectory such treatments are employed, the less likely they are to be effective and the more likely they will be burdensome. An example of this situation is using feeding tubes where there is evidence of efficacy only for those patients under consideration for a potentially curative surgical resection of their upper gastrointestinal cancers [3]. "CPM" may be described as all efforts to maintain comfort, dignity, and quality of life through the use of pain and symptom management, and generally, without the use of potentially life-prolonging measures. Many authors have dubbed this approach as "Comfort Care" [4] or "Comfort Measures Only" [5]. The prime goal and intent of CPM is to maximize comfort, dignity, and quality of life.

Healthcare is evolving from the traditional paternalistic medical model to a more autonomous and patient-directed model. Recently, this contemporary approach was termed "Patient-Centered Care" (PCC). According to the Picker Institute [6] and the Institute of Medicine [7], PCC is health care that is respectful of and responsive to individual patient preferences, expressed needs, and personal values [6-8]. PCC also emphasizes the need for effective communication, education, and information-sharing with patients and their family and friends, as well as facilitating integration of care and appropriate and timely transitioning. PCC promotes ethical health care that strives to improve patient satisfaction. Intrinsic to PCC is optimal communication between patients and clinicians. A recent systematic review concluded that patients vary in their preferences for communication style and in their desire for participation in decision making, thereby indicating the need for individualization [9]. Good communication between patients, their family members, and clinicians allows for exploration of values and goals of care, and promotes the discussion of preferences for the patient's future care. Written advance directives formalize these preferences and include living wills and designation of durable powers of attorney for health care, that specify health care proxies. There is great variability in the range and extent of intervention that patients with advanced cancer desire. When asked, some patients simply state "I want everything done" or "I want to fight until the end". Such statements should not simply be taken at face value, but should be further explored and discussed within the context of the patient's disease and associated prognosis [10]. Physicians, too, need to be clear about their statements to patients. For instance, the use of euphemisms to "soften the shock of bad news" may confuse or mislead patients [11].

The decision making process involved in healthcare may be divided into four major components: physician knowledge of patient medical history; physician disclosure of treatment choices; discussion of treatment choices; and selection of treatment choice [12]. Several models of decision making have been described. In the paternalistic model, physicians perform information management, assess options, and make treatment decisions for patients without consideration of patient preferences [13]. In the informed or consumer model, physicians provide all relevant information to their patients, and patients alone assess their options and make the final decision. The physician in this model serves as the technical expert to provide information and facilitate decisions made by a fully-autonomous patient [14]. The shared model essentially merges these two models into one where patients and physicians participate equally in all stages of decision making [15]. Moreover, the physician and other healthcare clinicians explore patient preferences and ensure that they are congruent with "evidence-based care" [16].

This study investigates the preferences of advanced cancer patients and their SDM's for commonly-offered active and aggressive medical therapies like cardiopulmonary resuscitation (CPR) & mechanical ventilation (MV), chemotherapy, antibiotics, anticoagulants, blood transfusions, feeding tubes and artificial hydration. This study serves to evaluate the correlations that exist between preferences for pursuing active and aggressive medical interventions and various patient characteristics, demographics, and other clinical parameters.

## Methods

### Study Population

A consecutive cohort of all new referrals to a regional palliative medicine program in Toronto, Canada was assembled prospectively between May 1, 2005 and June 30, 2006. Referrals included both cancer patients and patients with advanced non-cancer disorders. Patients were referred for consideration of palliative medical management and eventual end-of-life care. Patients were assessed either in their homes or in the hospital. This study focuses on the cancer patients. All patients or their substitute decision makers (SDM) provided consent to have their clinical data registered in a research database. The data collected was entered on a customized Microsoft™ Access 2007 database. This was done on an accrual basis.

### Measurement

All patients were examined within twenty-four hours of the initial referral. The initial consultation was designated as the baseline for the study. At baseline, basic demographic data was collected, the primary cancer diagnosis was recorded, and performance status was measured using the Palliative Performance Scale (PPSv2) [17]. PPSv2 measures performance status between 0% (patient dead) and 100% (patient completely functional) and is highly correlated with the Karnofsky Performance Status scale [18].

**Figure 1:**
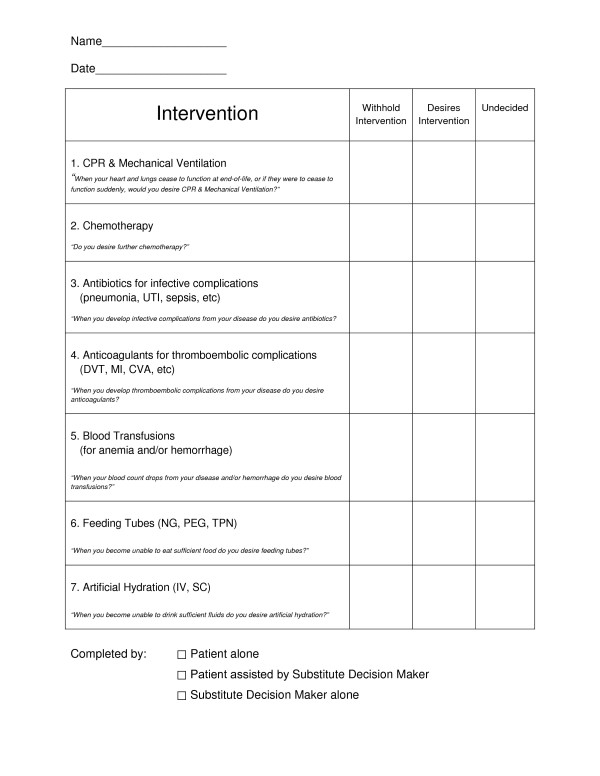
**Questionnaire to Assess Patient Preferences for Intervention**.

Upon completion of the history taking and physical examination, a questionnaire was administered (Figure 1), within the process of an interactive discussion with the patient and/or SDM. At the outset, it was ensured that the patient was mentally capable and fluent in English. In cases where the patient was mentally incapable the SDM was asked to express preferences. When English fluency was inadequate an interpreter was enlisted. The purpose of this questionnaire was to ascertain the patient's preferences for active interventions such as CPR together with MV, future chemotherapy, antibiotics for future infective complications, anticoagulants for future thromboembolic complications, blood transfusions for anemia and/or hemorrhage, feeding tubes and artificial hydration. The seven questions were discussed in a conversational manner while placing emphasis on the context of their diseases. Regarding CPR & MV, patients were informed of data that reflects less than 7% survival to discharge for advanced cancer patients suffering a cardio respiratory arrest [19]. The decisions regarding the listed interventions were recorded as "withhold intervention", "desires intervention", or "undecided". In the case of CPR & MV, cases that were "undecided" were defaulted into the "desires intervention" category. The rationale for this default position was that, in the event of cardiac and/or respiratory arrest, only the patients who specifically and definitively opted to withhold intervention should forego CPR & MV. If the patient or SDM inquired about patient life expectancy, a median life expectancy was quoted as estimated in relation to their baseline PPSv2 as follows: PPSv2 10-20 = median survival of 1 week; PPSv2 30-50 = median survival of 1 month; and PPSv2 ≥ 60 = median survival of 3 months [18]. This was justified by a recent study that showed improved satisfaction when prognostic data was provided [20]. Baseline preferences were not irrevocable as patient preferences were revisited as indicated by the needs of the patient or SDM. This acknowledges that preferences may vary over the disease trajectory [21].

The primary cancer diagnoses were classified as gastrointestinal (gastric, esophageal, small intestine, colorectal, biliary, pancreatic, liver), lung (non-small cell, small cell, mesothelioma), genitourinary (prostate, renal, bladder, ureter), breast, hematologic (all leukemias, lymphomas, myeloma), gynecologic (cervix, ovarian, uterine), and others (sarcoma, carcinoid, primary brain tumors, primary skin cancers, primary head and neck cancers, metastatic adenocarcinoma of unknown primary source).

After their baseline assessment, patients were treated in a supportive manner while respecting and complying with their expressed preferences, and were followed until their deaths. The Charlson Comorbidity Index (CCI), based upon parameters present at the time of referral, was calculated retrospectively according to published guidelines [22,23].

### Ethical Considerations

This study involved analysis of a palliative medicine database developed by the principal author. The database was anonymized and bears no links to patients. This study was approved by the research ethics board of the William Osler Health Centre in Toronto, Canada.

### Statistical Analysis

Only cancer patients (n = 380) were included in the study. Data was exported from the Microsoft™ Access 2007 database into S-Plus 6.2 for Windows for statistical analysis. For each intervention mutivariable logistic regression models were fitted to explore correlates of 1) uncertainty versus certainty in whether the intervention was wanted or not and 2) in those who were decided, desiring the intervention versus not desiring it. The following factors were considered as explanatory variables: the patient's age, gender (male, female), race (Caucasian, non-Caucasian), PPSv2, CCI and cancer type (gastrointestinal, lung, genitourinary, breast, hematologic, gynecologic, others) as well as the decision-maker regarding the interventions (patient alone, patient and SDM, SDM alone). Age, PPSv2 and CCI entered the models as continuous variables, Significance were assessed by fitting nested models and testing the drop in deviance with X^2 ^tests. A composite score reflecting the overall desire for AAMM was constructed by totaling the number of interventions desired by each patient. Any undecided entries were scored as 0.5 when doing the summing; this corresponds to assigning a probability of one-half to the event of an undecided intervention being accepted. Correlates of the composite score were explored using multivariable linear regression. For the modeling the composite score was expressed as a proportion out of 7 and the variance stabilizing transformation for proportions (inverse sine of the square root of the proportion) was applied to improve the statistical properties of the outcome variable. Predicted values from the model were obtained for selected variable values by setting the rest of the variables to their mean value (age = 72.9, ppsv2 = 50, CCI = 9) or to the most common value (gender = male, race = Caucasian, diagnosis = GI, decision maker= patient) and transforming back from the inverse sine square root transformation to proportions.

## Results

### Patient Characteristics

380 cancer patients were referred to the program during the study period. Slightly over half of the patients (54.5%) were male. The mean age at referral was 73 years (standard deviation 13 years, range 19 to 99). 76.6% of patients were 65 years or older while 23.4 were less than 65 years of age. The majority of referrals were Caucasian (86.3%). The most frequent primary cancer diagnoses were gastrointestinal (28.7%) and lung cancer (25.2%), followed by genitourinary (8.7%), breast (7.1%), gynecologic (5.8%) and hematologic (5.5%). 61.1% of patients presented with PPSv2 of greater than or equal to 50% while 38.9% presented with a PPSv2 of less than 50%. 59.2% of patients presented with a CCI of greater than or equal to 9 while 40.8% presented with a CCI of less than 9.

### Patient Certainty

At baseline, most patients and their SDM's were relatively secure about decisions pertaining to the seven interventions presented given that the rates of being "undecided" ranged from a high of 23.4% for chemotherapy to a low of 3.9% for feeding tubes (Table 1). In the multivariable models (Table 1), indecision was not related to the explanatory variables age, gender, race, PPSv2, CCI, cancer type or decision-maker for any of the interventions (all model P > 0.05) except for artificial hydration (model P = 0.037). Males were significantly more undecided about artificial hydration than females (odds ratio 2.61, P = 0.01), as were non-Caucasians versus Caucasians (odds ratio 2.44, P = 0.03).

**Table 1:** Correlates of being undecided versus decided regarding each intervention: multivariable logistic regression model results for each intervention.

	Chemo- Therapy N = 380	Anti- biotics N = 380	Anti- coagulants N = 380	Blood transfusion N = 380	Feeding tubes N = 380	Artificial hydration N = 380
Number (%) undecided about intervention	89 (23.4)	56 (14.7)	51 (13.4)	40 (10.5)	15 (3.9)	51 (13.4)

**Age**						
Odds ratio: per 10 yr increase	0.94	1.03	0.84	1.09	1.04	1.01
P-value	0.512	0.784	0.148	0.542	0.865	0.917
**Gender**						
Odds ratio: Male vs. Female	1.31	1.96	1.23	1.61	1.38	2.61
P-value	0.347	0.054	0.563	0.248	0.588	0.011
**Race**						
Odds ratio: Non- vs. Caucasian	0.75	1.77	1.46	1.81	1.67	2.44
P-value	0.462	0.168	0.374	0.214	0.481	0.034
**Cancer Diagnosis**						
Odds ratio:						
Lung vs. GI	1.2	0.97	0.97	1.9	1.62	1.04
GU vs. GI	1.29	0.54	0.98	1.38	1.21	1.1
Breast vs. GI	1.1	1.62	0.85	2.07	0	1.72
Hematologic vs. GI	2.23	2.06	0.89	1.8	2.25	2.56
GYN vs. GI	3.26	2.36	1.89	3.16	1.39	2.37
Other vs. GI	1.56	0.65	0.59	0.49	1.49	0.56
P-value	0.395	0.349	0.804	0.218	0.882	0.317
**PPSv2**						
Odds ratio: per 10 unit increase	0.91	1.01	0.77	0.99	1.07	0.83
P-value	0.433	0.964	0.083	0.971	0.801	0.214
**CCI**						
Odds ratio: per unit increase	0.92	0.89	0.9	0.96	0.83	0.9
P-value	0.104	0.066	0.123	0.553	0.073	0.117
**Decision maker**						
Odds ratio:						
Pat/SDM vs. Pat	1.09	1.69	1.21	1.7	1.2	1.7
SDM vs. Pat	1.42	1.28	0.5	1.22	2.8	0.82
P-value	0.701	0.445	0.137	0.504	0.498	0.177

Model P-value	0.147	0.291	0.430	0.491	0.688	0.037

### Patient Preferences

In decided patients, interventions that were most often desired by advanced cancer patients were: Anticoagulants (52.9%), Antibiotics (50.9%), Artificial Hydration (49.4%) and Blood transfusions (44.7%). Less frequently desired were Feeding tubes (34.0%), Chemotherapy (38.1%), and CPR/MV (25.5%). Table 2 shows the correlates of desiring each intervention. The overall model P-values were all significant, indicating that each model explained a statistically significant amount of the variation in each outcome.

**Table 2:** Correlates of desiring an intervention versus not desiring it: multivariable logistic regression model results for each intervention.

	CPR & MV* N = 380	Chemo- therapy N = 291	Anti-biotics N = 324	Anti-coagulants N = 329	Blood transfusions N = 340	Feeding tubes N = 365	Artificial hydration N = 329
Number (%) desiring intervention	97 (25.5)	111 (38.1)	165 (50.9)	174 (52.9)	152 (44.7)	124 (34)	162 (49.2)

**Age**							
Odds ratio: per 10 yr increase	0.67	0.68	0.72	0.74	0.72	0.72	0.7
P-value	< 0.0001	0.0007	0.0004	0.002	0.0002	0.0004	0.0001
**Gender**							
Odds ratio: Male vs. Female	1.33	1.58	1.37	1.49	1.29	1.81	1.38
P-value	0.324	0.145	0.241	0.138	0.339	0.032	0.237
**Race**							
Odds ratio: Non- vs. Caucasian	1.71	4.16	1.46	1.67	1.19	1.69	1.53
P-value	0.126	0.0004	0.310	0.172	0.613	0.131	0.255
**Cancer Diagnosis**							
Odds ratio: Lung vs. GI	1.25	0.86	0.81	0.99	0.82	1.19	0.73
GU vs. GI	0.62	0.71	1.03	1.26	1.17	0.9	1.13
Breast vs. GI	0.94	0.99	0.92	1.27	1.38	1.13	1.07
Hematologic vs. GI	0.72	0.47	0.45	0.42	0.66	0.4	0.28
GYN vs. GI	1.1	1.55	1.06	0.85	1.05	1.53	1.06
Other vs. GI	0.64	0.6	0.73	0.69	0.79	1.6	0.74
P-value	0.632	0.795	0.878	0.627	0.882	0.510	0.494
**PPSv2**							
Odds ratio: per 10 unit increase	1.32	1.97	1.45	1.55	1.34	1.43	1.47
P-value	0.021	< 0.0001	0.001	0.0001	0.007	0.002	0.0007
**CCI**							
Odds ratio: per unit increase	1.09	1.17	1.08	1.05	1.09	1.13	1.08
P-value	0.113	0.016	0.153	0.294	0.077	0.016	0.128
**Decision maker**							
Odds ratio:							
Pat/SDM vs.Pat	1.89	3.65	1.48	1.84	1.65	2.92	1.87
SDM vs. Pat	1.7	4.24	2.34	2.98	1.84	2.76	2.84
P-value	0.212	0.003	0.134	0.031	0.239	0.007	0.040

Model P-value	0.0001	< 0.0001	0.0001	< 0.0001	0.002	< 0.0001	< 0.0001

* Cardiopulmonary Resuscitation & Mechanical Ventilation

74.5% of decided patients expressed the desire to withhold CPR & MV. Patients who desired this intervention tended to be younger (odds ratio 0.67 for every ten year increase in age, P < 0.0001) and to have a higher performance status (odds ratio 1.32 for every ten units increase in PPSv2, P = 0.021).

61.9% of decided patients expressed the desire to withhold further chemotherapy if offered. Patients who desired this intervention tended to be younger (odds ratio 0.68 per ten year increase in age, P = 0.0007), to be non-Caucasian (odds ratio 4.16, P = 0.0004), to have a higher performance status (odds ratio 1.97 for every ten units increase in PPSv2, P < 0.0001), to have higher CCI (odds ratio 1.17 for every unit increase, P = 0.016) and to have a SDM involved in the decision (odds ratios 3.65 and 4.24 for patient/SDM and SDM alone, P = 0.003).

49.1% of decided patients expressed the desire to withhold future antibiotic therapy. Patients who desired this intervention tended to be younger (odds ratio 0.72 for every ten year increase in age, P = 0.0004) and to have a higher performance status (odds ratio 1.45 for every ten units increase in PPSv2, P = 0.001).

41.7% of decided patients expressed the desire to withhold future anticoagulant therapy. Patients who desired this intervention tended to be younger (odds ratio 0.74 per ten year increase in age, P = 0.002), to have a higher performance status (odds ratio 1.55 for every ten units increase in PPSv2, P = 0.0001) and to have a SDM involved in the decision (odds ratios 1.84 and 2.98 for patient/SDM and SDM alone, P = 0.031).

55.3% of decided patients expressed the desire to withhold future blood transfusions. Patients who desired this intervention tended to be younger (odds ratio 0.72 for every ten year increase in age, P = 0.0002) and to have a higher performance status (odds ratio 1.34 for every ten units increase in PPSv2, P = 0.007).

66% of decided patients expressed the desire to withhold the future insertion of a feeding tube. Patients who desired this intervention tended to be younger (odds ratio 0.72 for every ten year increase in age, P = 0.0004), to be male (odds ratio 1.81, P = 0.032), to have a higher performance status (odds ratio 1.43 for every ten units increase in PPSv2, P = 0.002), to have a higher CCI (odds ratio 1.13 for every unit increase, P = 0.016) and to have a SDM involved in the decision (odds ratios 2.92 and 2.76 for patient/SDM and SDM alone, P = 0.007).

50.8% of decided patients expressed the desire to withhold artificial hydration. Patients who desired this intervention tended to be younger (odds ratio 0.70 for every ten year increase in age, P = 0.0001), to have a higher performance status (odds ratio 1.47 for every ten units increase in PPSv2, P = 0.0007) and to have a SDM involved in the decision (odds ratios 1.87 and 2.84 for patient/SDM and SDM alone, P = 0.04).

### Composite Score

The composite score reflects the number of interventions desired by each patient, counting undecided as 0.5. There was a tendency towards the extremes in the composite score, with 41.6% of patients wanting one or fewer interventions and 23.9% wanting six or more interventions. Table 3 shows the multivariable regression results for the transformed composite score. Overall the model was significant (model P < 0.0001, R^2 ^= 14.2%). The number of interventions desired decreased with increasing age (P < 0.0001), was higher for non-Caucasians than Caucasians (P = 0.042), increased with increasing PPSv2 and was greater when a SDM was involved in the decision (P = 0.027). Non-statistically significant trends to prefer more AAMM was observed with male gender (p = 0.077) and higher levels of the Charlson Comorbidity index (p = 0.059). The patient's cancer diagnosis did not demonstrate any statistically significant trends with respect to a preference for more interventions.

**Table 3:** Correlates of the composite score reflecting the overall desire for AAMM. The composite score is the number of interventions desired by each patient, scoring any undecided entries as 0.5. For modeling purposes the score was expressed as a proportion out of seven and a variance stabilizing transformation applied.

Explanatory variable	Regression Coefficient	P-value	Predicted Proportion of Interventions Desired*
**Age**	-0.09 per 10 year increase	< 0.0001	
30 years			0.69
40 years			0.60
50 years			0.51
60 years			0.42
70 years			0.34
80 years			0.25
**Gender**		0.077	
Male	0.107		0.31
Female	reference		0.22
**Race**		0.042	
Caucasian	reference		0.31
Non-Caucasian	0.162		0.47
**Cancer Diagnosis**		0.905	
GI	reference		0.31
Lung	-0.001		0.31
GU	-0.022		0.29
Breast	0.022		0.33
Hematologic	-0.116		0.21
GYN	0.040		0.35
Other	-0.075		0.24
**PPSv2**	0.095 per 10 unit increase	0.0002	
20			0.09
30			0.15
40			0.23
50			0.31
60			0.40
70			0.50
**CCI**	0.021 per unit increase	0.059	
7			0.27
9			0.31
11			0.35
**Decision maker**		0.027	
Patient	reference		0.31
Patient/SDM	0.173		0.48
SDM	0.245		0.55

*For each variable shown, predicted proportions were obtained by setting the rest of the variables to their mean value (continuous variables) or most common value (categorical variables) and back-transforming.

## Discussion

This is the first study to evaluate the preferences of patients with advanced cancer referred to a palliative medicine consultative program for a group of seven common active and aggressive interventions while en route to their eventual death. Overall, there was a trend to desire a mostly-conservative palliative approach, as nearly 42% of patients desired one or fewer interventions. At baseline, most patients and their SDM's were relatively secure about decisions pertaining to the seven interventions presented, given that the rates of being "undecided" ranged from a high of 23.4% for chemotherapy to a low of 3.9% for feeding tubes.

This study demonstrated that older age was associated with a greater tendency to withhold each of the potentially life-prolonging measures offered. This is consistent with previously published reports [24,25]. One study showed that younger patients suffering from advanced cancer, with dependent children, are more likely to desire aggressive management, within an acute hospital setting [26]. Unfortunately, this was associated with reduced quality of life in their last week of life [26].

Male gender was associated with a greater tendency to desire feeding tubes but not the other active and aggressive measures offered. However, the suggestion of gender disparity in this study is consistent with a previously published report of a mixed (cancer and noncancer) cohort of patients with advanced illness [4]. A literature search conducted on Ovid MEDLINE, PubMed, The Cochrane Library, CINAHL, and HealthSTAR databases between 1997 and July 2010 failed to locate reports that focused on solely on cancer.

This study demonstrated that non-Caucasian patients were more desirous of chemotherapy and they tended to desire more interventions overall according to the composite score analysis. A recent oncology study demonstrated that more Black patients (45%) and Hispanic patients (34%) desired potentially life-prolonging measures than White patients (14%) even if they had only a few days to live [27]. Another study involving a combination of cancer and noncancer terminal illnesses also showed that more Black patients (28%) and Hispanic patients (21.2%) than white patients (15%) desired potentially life-prolonging drugs, even if they caused them to "feel worse all the time" [28]. In the same study, fewer Black patients (49%) and Hispanic patients (57%) than White patients (74%) desired "potentially life-shortening palliative drugs" [28]. Another study demonstrated that Blacks and Hispanics are less likely to acknowledge and accept their terminal illness [27] and Blacks express greater discomfort when discussing death [29]. In one study, Blacks were less likely than Caucasians to complete an advance directive (35.5% vs. 67.4%, p < 0.001), and they tended to have less favourable beliefs about hospice care as well as a general mistrust of the health care system [29].

Although our study did not assess the religion or spiritual correlates of patient preferences, a recent study demonstrated that higher levels of religiousness was associated with desiring all aggressive measures (OR 1.96; 95% CI 1.08-3.57) with the intent to prolong life [30]. In addition, African-Americans and Hispanics rated religion as important more frequently than Caucasians [30].

Performance status, as measured with PPSv2, was highly-correlated with preferences. For each intervention higher PPSv2 was significantly associated with increased odds of desiring the intervention and it was also associated with higher composite scores. These results are intuitive as patients with higher PPSv2 are more functional, less dependent, and also have a longer survival period relative to patients with lower levels of PPSv2. A recent systematic review confirmed that PPSv2 is highly correlated with survival in cancer patients and is useful in establishing prognoses [31].

Comorbid illness as measured with the Charlson Comorbidity Index (CCI) was associated with a greater desire for chemotherapy and feeding tubes. These trends may seem paradoxical as one might expect that those patients with the highest CCIs are among the "sickest" and thus more likely to adopt less AAMM. A potential explanation may be that such patients are so accustomed to receiving medical "micromanagement" addressing all of their comorbid factors that they have a propensity to desire AAMM, perhaps, based on a conditioned response.

The involvement of Substitute Decision Makers (SDM) in establishing preferences for active and aggressive intervention was associated with greater desire to pursue AAMM. The composite intervention analysis (Table 3) demonstrated that this trend was greater when SDMs acted alone versus SDMs assisting the patient. The individual intervention analysis (Table 2) showed a greater tendency for AAMM when the SDM acted alone versus SDMs assisting the patient for all interventions except chemotherapy and feeding tubes. Although the explanation of this phenomenon is unclear, it may rest with the supposition that SDMs base such decisions on what they might decide for themselves at that instant [32]. Given that SDMs are generally healthier than their patient counterparts, it is more likely that they will elect for a more active and aggressive mode of care [32].

This study has a number of weaknesses. Data was collected from a single palliative program and employed a questionnaire (Figure 1) that had not undergone reliability or validity studies. It only considered preferences regarding withholding active and aggressive interventions while not adjusting for those patients who were receiving those interventions at baseline nor those that later decided to withdraw active interventions. In addition, there was no data on socioeconomic status or stratification for the different forms of the individual interventions. Although there was no randomization, this study was nonetheless based on a large prospective and sequential case series.

## Conclusion

Exploring patient preferences for active intervention is central and intrinsic to delivering "Patient-Centered Care" that is consistent with the ethical principles of informed consent and autonomy [33]. Futile treatments remain highly utilized among advanced cancer patients and are associated with significant implications for healthcare economics [34]. Allowing patients to express their preferences, has the potential to reduce the volume of futile treatments, thus promoting financial sustainability of our health care systems while addressing the ethical principle of distributive justice [33].

This study exemplifies the first stage of shared decision making process, namely, establishing patient treatment preferences. This information is vital and foundational in the development of advance directives as well as allowing for optimal planning for the patient's future healthcare. This study demonstrates that the majority of advanced cancer patients prefer to adopt CPM over AAMM. Therefore, it behooves physicians and other healthcare professionals to explore the preferences for active and aggressive intervention among their patients suffering from all forms of advanced illness, including those with cancer. It should never be assumed that patients desire continuation of active and aggressive interventions for the balance of their lives. Moreover, patients and their SDMs should be counseled around the fact that although active and aggressive measures may be beneficial and potentially life-prolonging during the earlier phases of their malignant disease, they generally become increasingly ineffective, burdensome, and futile later in the disease trajectory. Hopefully, this information may be elicited at an earlier stage rather than waiting for the patient to be referred for palliative medical management. Moreover, eliciting this information at an earlier stage may enhance the likelihood that the patient will articulate their own unassisted views on treatment preferences. Finally, the determination of clearly articulated goals and objectives of care may facilitate earlier transition to a completely palliative mode of care with the potential for improvements in comfort, dignity, and quality of life.

## Competing interests

The authors declare that they have no competing interests.

## Authors' contributions

Conception and design was carried out by VM and JP. Collection and assembly of data was carried out by NB and ARM. Data analysis was carried out by JP and ME. Manuscript writing and final approval of manuscript was carried out by VM, JP, ME, NB, and ARM.

## Pre-publication history

The pre-publication history for this paper can be accessed here:

http://www.biomedcentral.com/1471-2407/10/592/prepub
